# Novel Slide-Ring Material/Natural Rubber Composites with High Damping Property

**DOI:** 10.1038/srep22810

**Published:** 2016-03-07

**Authors:** Wencai Wang, Detao Zhao, Jingna Yang, Toshio Nishi, Kohzo Ito, Xiuying Zhao, Liqun Zhang

**Affiliations:** 1 Key Laboratory of Beijing City on Preparation and Processing of Novel Polymer Materials, Beijing University of Chemical Technology, Beijing 100029, PR China; 2 Beijing Engineering Research Center of Advanced Elastomer, Beijing, PR China; 3 Engineering Research Center of Elastomer Materials Energy Conservation and Resources, Ministry of Education, PR China; 4 Department of Organic and Polymeric Materials, Graduate School of Science and Engineering, Tokyo Institute of Technology, 2-12-1 O-okayama, Meguro-ku, Tokyo 152-8552, Japan; 5 Graduate School of Frontier Sciences, The University of Tokyo, 5-1-5 Kashiwanoha, Kashiwa, Chiba 277–8561, Japan

## Abstract

A novel class of polymers called “slide-ring” (SR) materials with slideable junctions were used for high damping composites for the first time. The SR acts as the high damping phase dispersed in the natural rubber (NR) matrix, and epoxidized natural rubber (ENR) acts as the compatibilizer. The morphological, structural, and mechanical properties of the composites were investigated by atomic force microscope (AFM), transmission electron microscope (TEM), dynamic mechanical thermal analyzer (DMTA), rubber processing analyzer (RPA), and tensile tester. AFM and TEM results showed that the SR phase was uniformly dispersed in the composites, in a small size that is a function of ENR. DMTA and RPA results showed that the damping factor of the composites is much higher than that of NR, especially at room temperatures. Stretch hysteresis was used to study the energy dissipation of the composites at large strains. The results showed that SR and ENR can significantly improve the dissipation efficiency at strains lower than 200% strain. Wide-angle X-ray diffraction was used to study the strain-induced crystallization of the composites. The results indicated that the impact of the SR on the crystallization of NR is mitigated by the insulating effect of ENR.

Earthquake is an unpredictable natural disaster with the characteristics of high incidence and great destruction. Improving the anti-seismic strength of buildings is an effective approach to protect life and reduce economic loss. Isolation bearing is an important device to reduce the seismic force transmitted to buildings with functions of restoring force and energy dissipation^1^,^2^. Furthermore, it increases the structure deformability and hysteretic damping ability of buildings. It also reduces the seismic influence coefficient. The materials used for isolation bearings must be strong in the vertical direction and flexible in the horizontal direction to resist damage^3^,^4^,^5^,^6^.

Due to the unique ability for strain-induced crystallization, NR can crystallize at high deformations^7^,^8^,^9^. The crystallization of NR can increase its tensile strength and ability to resist damage. All these desirable mechanical properties of NR are important for the requirements of isolation bearings. But the macromolecular chains of natural rubber are flexible, which causes less internal friction between the macromolecules. The less internal friction would affect the damping property seriously. Many additional measures such as lead core technology and damper devices are essential for energy dissipation^6^. On the other hand, however, the additional measures will increase the cost of the rubber bearing, and the leak of lead is environmentally harmful. Thus, improving the damping property of natural rubber is an effective way to avoid the above shortcomings.

Up to now, a variety of methods have been reported for the preparation of high damping rubbers^10^,^11^,^12^,^13^,^14^. Notable methods include the chemical modification and physical blending. Wu *et al.* mixed hindered amine and hindered phenol with chlorinated polyethylene and polyacrylate rubber to prepare high damping organic hybrid materials. There are two damping peaks in the dynamic mechanical spectra, one for the glass transition of the matrix and the other for the hydrogen bond network^10^,^11^,^12^. By using hindered phenol (AO-80), Zhao *et al.* prepared high damping carboxylated nitrile rubber with a high damping peak and a controllable peak position by the effect of hindered phenol(AO-80)^13^. Tian *et al.* studied the influence of magnetic particles on the damping property of nitrile butadiene rubber. They showed that the permanent magnetic rubber composites have high loss factors in a wide frequency range^14^. In terms of mechanical strength and damping property, most rubber composites do not meet the requirements for isolation rubber bearings.

In recent years, slide-ring materials have drawn considerable attention because of their supramolecular architecture with topological characteristics^15^,^16^,^17^. The SR has a poly(ethylene glycol) (PEG) backbone with many α-cyclodextrins (α-CD) crossed on. The α-CDs are modified by polycaprolactone (PCL). The bulky adamantine groups at both ends of the main chains are introduced to prevent the dissociation of the cyclic molecules^18^,^19^,^20^,^21^,^22^. The slide-ring materials are obtained by the crosslinking of PCL. The key difference of SR from conventional rubbers is that the crosslinking junctions on α-CDs can freely slide along the backbone. This phenomenon, which is called the “pulley effect”, is illustrated in Fig. 1(a). Uncrosslinked free rings show an air suspension effect when the mutual distance between the rings is compressed by external deformations^18^,^23^,^24^,^25^. Because of these effects, SR have distinguished properties of low compression set, small stress relaxation, and large loss factor in a wide frequency range. This novel material with “pulley effect” has already been applied to coating materials for automobiles^18^.

Considering the large loss factor of SR, the introduction of SR into NR should be an effective way to improve the damping property of NR. To the best of our knowledge, the use of SR in damping rubber composites has not been reported. In this blends, the SR is acting as damping phase to provide the damping property needed in dissipating seismic energy, while the NR matrix is to provide the high strength needed in large deformations. The design concept of high damping composites is illustrated in Fig. 1(b). Considering the abundance of -OH in SR, the SR phase can be more easily dispersed in polar polymers than in non-polar polymers like NR. To achieve a better interfacial compatibility between SR and NR phase, the utilization of a compatibilizer is desirable. Epoxidized natural rubber (ENR) is produced by epoxidizing natural rubber with peracetic acid and has a good compatibility with NR^26^,^27^,^28^,^29^. On the other hand, the polar epoxy groups in ENR has a good compatibility with SR, which makes ENR a good compatibilizer for slide-ring/natural rubber composites. In this work, slide-ring/natural rubber composites were prepared for high damping applications. The morphological, structural, and mechanical properties of the natural rubber/slide-ring composites were systematically investigated. Strain-induced crystallization in the slide-ring/natural rubber composites was studied by using synchrotron wide angle X-ray diffraction.

## Experimental

### Materials

The natural rubber (smoked sheet) was purchased from Nanjing Shengdong Chemical Co., Ltd. The slide-ring material (SeRM Elastomer SH3400S) is provided by Advanced Soft Materials, Inc. (Tokyo, Japan). The molecular weight of the PEG used in the SR is 35 000. There are ca. 103 α-CDs molecules per PR molecule, corresponding to an inclusion ratio of 26%. The PR was modified by PCL and had a weight average molecular weight of ca. 600 000. Epoxidized natural rubber(ENR) was supplied by Agricultural Products Processing Research Institute, Chinese Academy of Tropical Agricultural Sciences (Zhanjiang, Guangdong). Other compounding ingredients, such as sulfur, zinc oxide, stearic acid, N-Cyclohexyl-2-benzothiazole, 2,2′-Dibenzothiazole disulfide, and tetramethyl thiuram disulfide, were purchased from Shenghua Rubber and Plastic Products Co., Ltd. and used as received.

### Preparation of natural rubber/slide-ring composites

First, the SR was placed in an oven for 2 h at 175 °C to make sure that the crosslinking agent could sufficiently crosslink the PCL side chains. The formulation used for the natural rubber/slide-ring composites is shown in Table 1. The NR and ENR were plasticated in a two-roll mixer for 1 min before SR was added. Other compounding ingredients were mixed in the two-roll mixer at a front roll speed of 45 r/min and a back roll speed of 32 r/min. The total processing time was completed within 10 minutes. The compounds were further vulcanized by compression molding at 145 °C and 15 MPa. The binary and ternary slide-ring material/natural rubber composites will be denoted in the subsequent discussion as NR/SR (100/z) and NR/ENR/SR (x/y/z), respectively, where x stands for the percentage of NR, y stands for the percentage of ENR, and z stands for the phr of SR.

### Characterization methods

AFM images were recorded by a multimode bruker. All the images were obtained in the contact mode with a scanning angle of 90° to the long axis of the cantilever beam at a scanning velocity of 1 Hz. A standard V-shaped silicon nitride cantilever with a square-pyramidal tip was used.

The TEM images were obtained by transmission electron microscopes (H-800 and JEM-3010, Hitachi, Japan). The test samples were obtained by ultra-thin sectioned.

The mechanical properties were measured according to ASTM D638 on a CMT4104 electronic tensile tester (SANS, China) at a crosshead speed of 500 mm/min. The dumbbell-shaped samples (25 mm × 6 mm × 2 mm) were prepared according to ISO/DIS 37-1990. The mechanical properties data were the mean value of five times measurements. The hysteresis test data were the average of the results obtained from five samples under the same conditions.

Dynamic mechanical measurements were carried out on a dynamic mechanical analyzer (DMAVA3000, 01 dB Co., Ltd., France). The specimens were 15 mm long, 6 mm wide, and about 2 mm thick. The temperature dependence of the loss factor (tan δ) was measured in the temperature range −100 to 100 °C at a frequency of 10 Hz, a deformation of 0.1%, and a heating rate of 3 °C/min.

A rubber process analyzer (RPA 2000, Alpha Technologies, USA) was employed to study the damping change of the rubber composites. A dynamic strain sweep was conducted first by varying the strain from 1% to 200% at a constant frequency of 1 Hz at 30 °C. Dynamic frequency sweeps were then carried out from 0.1 to 10 Hz at a constant strain of 5% at 30 °C. The experimental data is the average of the results obtained from five samples under the same conditions. Each error bar indicates the standard deviation.

Synchrotron WAXD experiments of the NR/SR composites were carried out at room temperature at the BL16B1 beamline in Shanghai Synchrotron Radiation Facility, Shanghai, China. The wavelength used in BL16B1 was 0.124 nm, and the distance between the sample and the detector was 137.2 mm. The two-dimensional (2D) WAXD patterns were recorded every 50 s by a Mar CCD 165 X-ray detector system. The drawing measurements were performed at 25 °C. Each sample (100 × 10 × 1 mm^3^) was mounted between the two clamps of a homemade manual tester. The experimental data is the average of the results obtained from three samples under the same conditions. Each error bar indicates the standard deviation.

## Results and Discussion

### Microscopic morphology of slide-ring material/natural rubber composites

**Figure 2:**
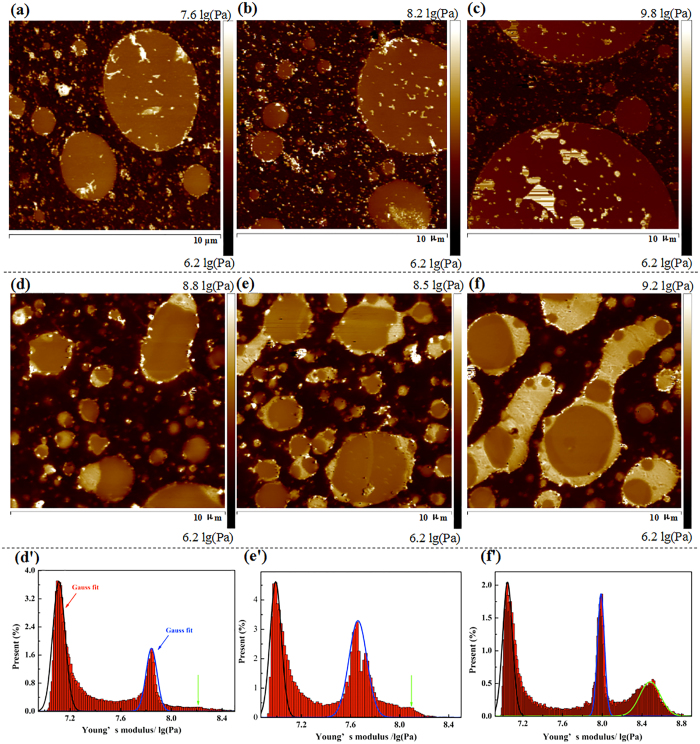
AFM images of (**a**) NR/SR (100/30), (**b**) NR/SR (90/40), (**c**) NR/SR (90/50), (**d**) NR/ENR/SR (90/10/40), (**e**) NR/ENR/SR (80/20/40), and (**f**) NR/ENR/SR (70/30/40) composites.

Figure 2 shows the AFM images for the polished surface of the natural rubber/slide-ring composites. The NR matrix and SR phase can be clearly distinguished in Fig. 2(a–c). The dark matrix is assigned to natural rubber, while the bright domains are assigned to the SR phase. The largest particles of the dispersed SR phase are about 5–10 μm in diameter, and the diameter increases with increasing amount of SR. There also show a clear interface between the SR phase and the NR matrix, indicating the fact that there is no strong interfacial bonding between the two phases. The reason is due to that the NR matrix is non-polar, while the SR phase is polar with many polar intermolecular hydrogen bonds.

Figure 2(d–f) shows the surface of the NR/ENR/SR (90/10/40), NR/ENR/SR (80/20/40), and NR/ENR/SR (70/30/40) composites. The ENR phase can be observed as the brightest region in the images of the ternary composites. ENR is mainly distributed at the interface of the SR and NR phases. Under the effect of ENR, the SR phase was uniformly dispersed, with diameters of 2–3 μm, indicating that ENR is a good compatibilizer for natural rubber/slide-ring composites. In the NR/ENR/SR (90/10/40) composite, the ENR phase is scattered around the SR phase. The SR phase is completely coated by the distinct ENR phase, as shown in Fig. 2(e,f). The ENR phase improves the interfacial compatibility of SR and NR by covering the surface of the dispersed phase. This microstructure, which contributes to the dispersion of the SR phase is called “elastic-coating”, as shown in Fig. 2(d–f).

The histogram in Fig. 2(d’–f’) shows statistical results of the Young’s modulus for samples NR/ENR/SR (90/10/40), NR/ENR/SR (80/20/40), and NR/ENR/SR (70/30/40). The statistical distribution of the Young’s modulus can be well-described by a Gaussian function with a mean value of 7.05 lg(Pa) for NR, 7.71 lg(Pa) for SR, and 8.26 lg(Pa) for ENR. The SR phase is soft and sticky due to the low molecular weight, and the affects of viscosity to the accuracy of AFM probe can not be ignored. As a result, the Young’s modulus of SR calculated by AFM data is higher than NR which is an opposite trend compared with the mechanical properties.

**Figure 3:**
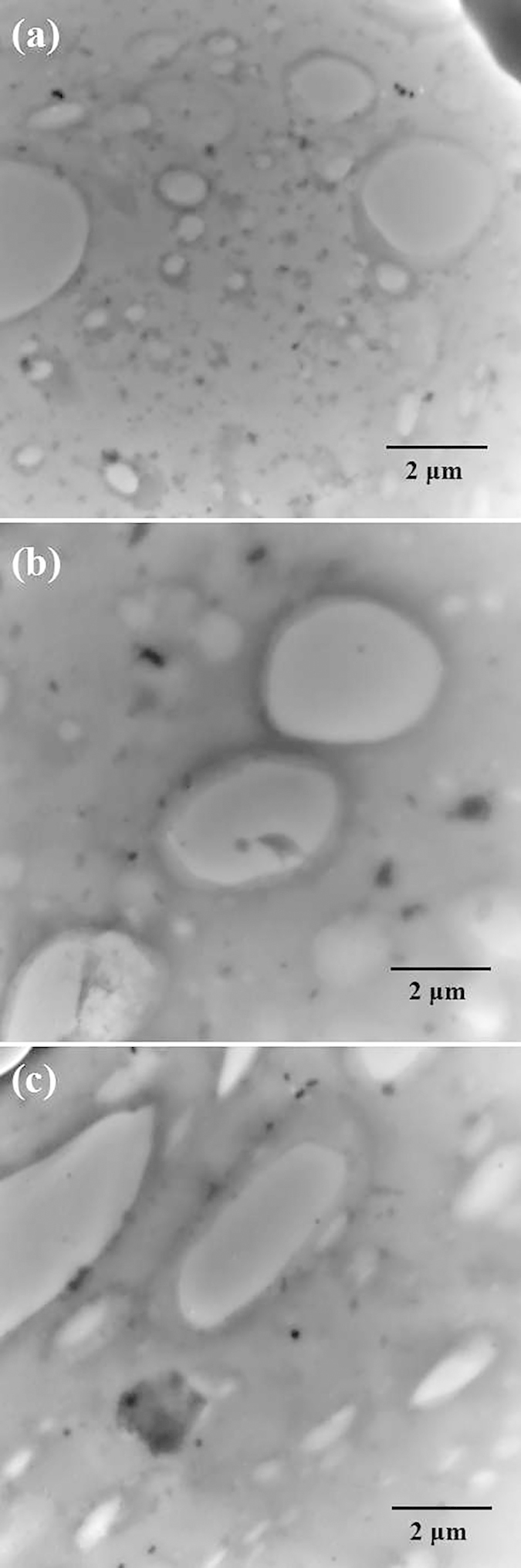
TEM images of ternary NR/ENR/SR composites: (**a**) NR/ENR/SR (90/10/40), (**b**) NR/ENR/SR (80/20/40), and (**c**) NR/ENR/SR (70/30/40).

This “elastic-coating” structure in the ternary NR/ENR/SR composites can be further confirmed by the TEM images shown in Fig. 3. It can be observed in Fig. 3 that the brighter SR phase with a maximum diameter of 3 μm is uniformly distributed in the NR matrix. Furthermore, the ENR phase used as the compatibilizer is distributed at the interface of the SR and NR phases. The “elastic-coating” structure mentioned above can be seen in these TEM images. With increasing content of the ENR phase, the “elastic-coating” structure becomes more pronounced, and the boundary phase becomes thicker, showing the same trends by the AFM images. The microscopic morphology results confirm the existence of the “elastic-coating” structure in the ternary NR/ENR/SR composites. This “elastic-coating” structure may provide a new design concept for the preparation of micro-controllable composites.

### Mechanical properties of natural rubber/slide-ring composites

#### Tensile properties

The effect of SR and ENR on the tensile strength (TS) and elongation at break (EB) of natural rubber/slide-ring composites is presented in Table 2. The tensile strengths of the binary NR/SR composites are moderately lower than that of NR because of the low tensile strength of SR. The tensile strengths of the ternary NR/ENR/SR composites are higher than NR/SR(100/40) composite because of the “elastic-coating” microstructure in the ternary NR/ENR/SR composites. This structure promotes the interaction between the SR phase and NR matrix. The tensile modulus of SR/NR composites decrease with increasing content of SR. In other words, the addition of SR makes the NR/SR composites softer. The effect of the SR phase on the tensile modulus at 100% and 300% strains of the composites is significant (up to 50% reduction from those of NR). The large deformations of the SR/NR composites under low stresses makes these composites as good rubber isolation bearing materials.

Elongations at break higher than 500% and tensile strengths higher than 10 MPa are required for isolation rubber bearings^30^. There are a large amount of polar groups existed in SR phase and they can absorb the accelerators and activating agents used in the curing process of NR. This process will reduce the cross link density of natural rubber. The elongation at break of natural rubber/slide-ring composites is 10% longer than NR. The elongations at break of the composites are higher than 770%, and their tensile strengths are higher than 12 MPa. In general, the natural rubber/slide-ring composites can meet the ISO standard for isolation rubber bearings^30^.

#### DMTA responses

**Figure 4:**
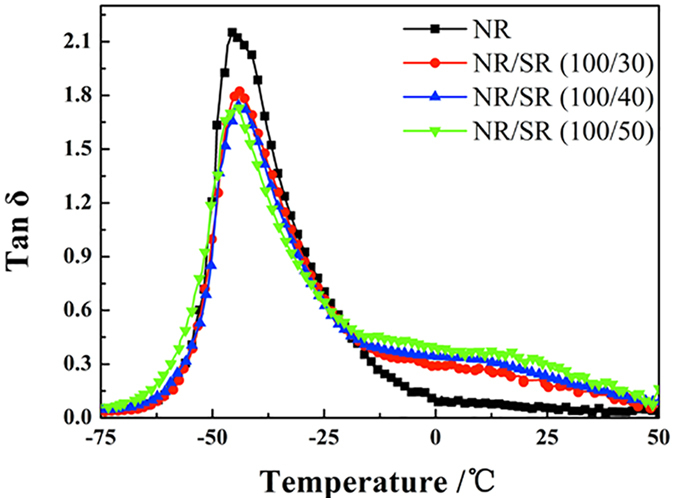
Temperature dependence of tan δ for binary NR/SR composites.

Figure 4 shows the temperature dependence of the loss factor, tan δ, for the binary NR/SR composites. A high and broad tan δ peak is essential for good damping performance. For each composite, only one broad tan δ peak is observed. The value of tan δ at room temperature is significantly higher for SR/NR than for NR. The value of tan δ at room temperature is the highest for the NR/SR (100/50) composite, followed by those for the NR/SR (100/40) composite, the NR/SR (100/30) composite, and NR. The amount of SR in the NR/SR composites follows the same order. This indicates that SR can improve the damping property of composites with “pulley effect”.

**Figure 5:**
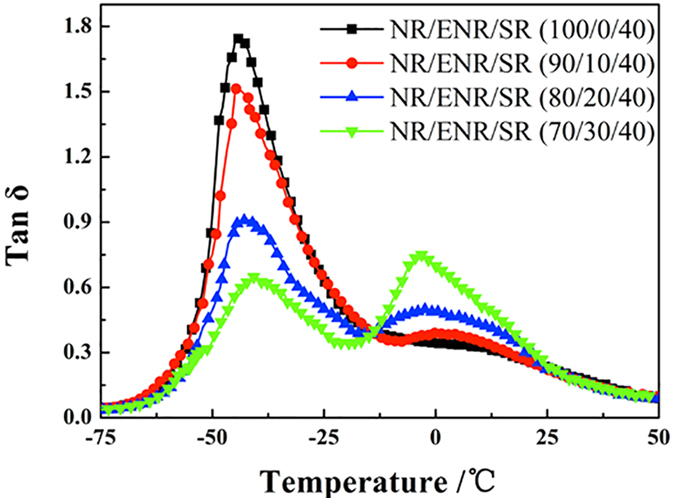
Temperature dependence of tan δ for ternary NR/ENR/SR composites.

Figure 5 shows the temperature dependence of loss factor, tan δ, for the ternary NR/ENR/SR composites. Two damping peaks are clearly observed at ENR contents higher than 20%. One peak can be assigned to the NR-rich domain and the other to the ENR-rich domain. With increasing ENR content, the height of the first damping peak decreases significantly, but the height of the second peak increases gradually. The ternary NR/ENR/SR composites show a broader efficient damping temperature range at room temperatures than the binary NR/SR composites. ENR is not only an interfacial compatibilizer to promote the interaction between the SR phase and the NR matrix, but also a damping phase to improve the height of tan δ at room temperatures where the Tg of ENR is located. So it is reasonable to conclude that tan δ increases with increasing ENR content.

### Variation of damping factor

For isolation rubber bearings, tan δ values higher than 0.1 (equivalent damping ratio of 10%) in the 0.2–5 Hz frequency range and 10–200% strain range are required, according to ISO standard^30^.

**Figure 6:**
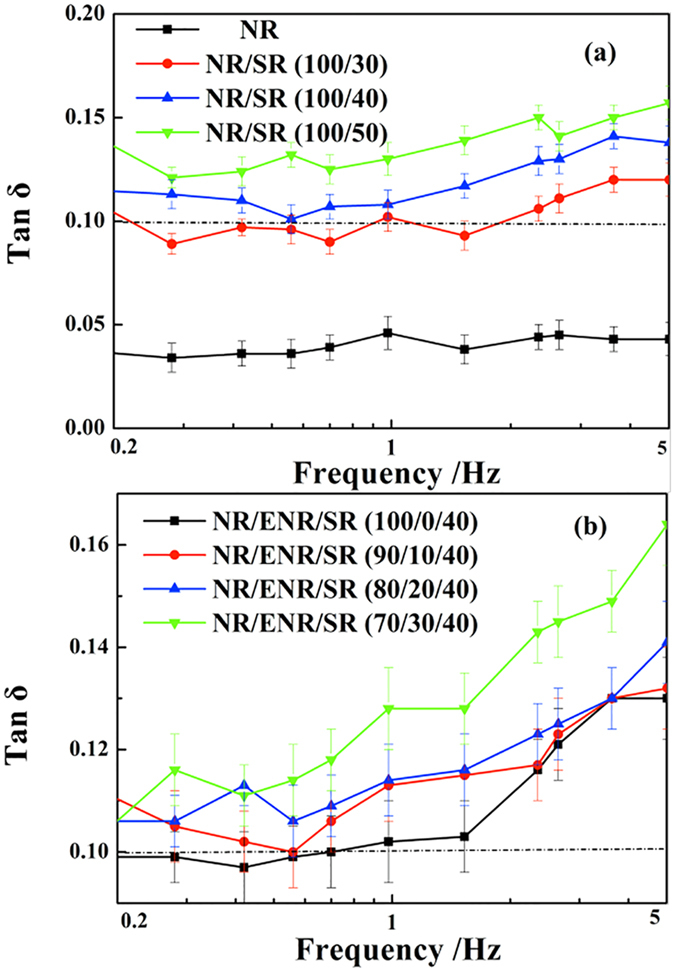
Variation of tan δ with dynamic frequency from 0.1 to 10 Hz at 5% strain for (**a**) binary NR/SR composites and (**b**) ternary NR/ENR/SR composites.

Figure 6 shows the variation of tan δ with dynamic frequency for the composite materials. The tan δ values of both the binary NR/SR composites and ternary NR/ENR/SR composites increase with the increasing of SR or ENR content. The tan δ values are higher than 0.1 in the frequency range 1–5 Hz for NR/SR(100/40), NR/SR(100/50) and ternary NR/ENR/SR composites. In the frequency range 0.2–1 Hz, only the NR/SR (100/50) composite can exceed ISO standards, and the tan δ values of the other composites fluctuate at the 0.1 level. These results show that the NR/SR composites have better damping performance at high frequencies. And the low tan δ values at low frequencies can be improved by adjusting the SR and ENR contents.

**Figure 7:**
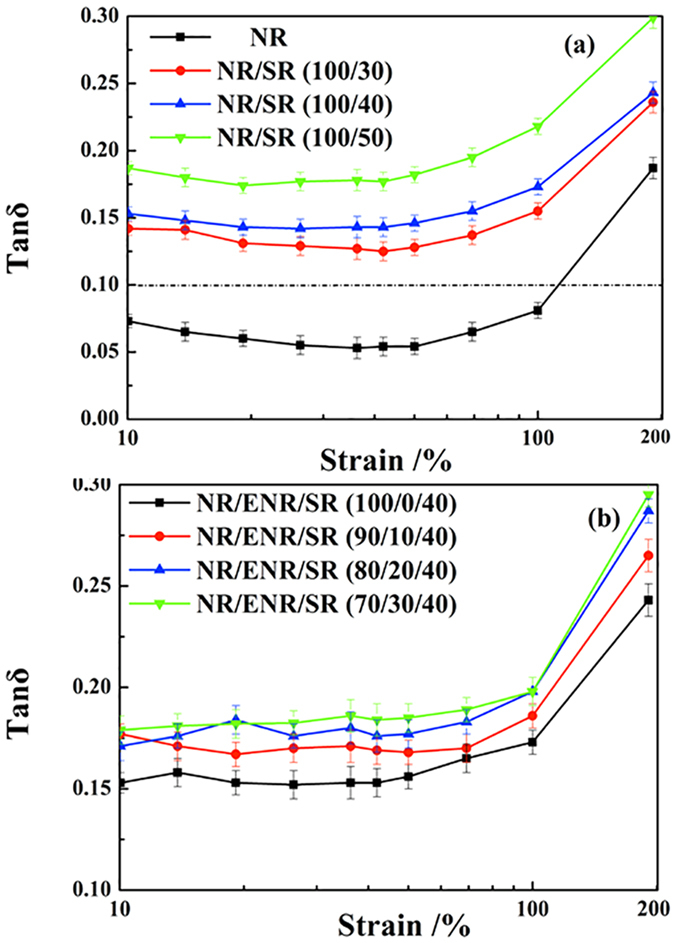
Variation of tan δ with dynamic strain from 1% to 200% at 1 Hz for (**a**) binary NR/SR composites and (**b**) ternary NR/ENR/SR composites.

Figure 7 shows the variation of tan δ with dynamic strain. The value of tan δ increases with the increase of strain. The tan δ values of both the binary NR/SR composites and ternary NR/ENR/SR composites should be higher than 0.1 in the strain range 10–200% to meet the standards for isolation rubber bearings. At a strain of 100%, the tan δ of the NR/ENR/SR (70/30/40) composite reaches 0.2, while that of NR is less than 0.1. Overall, these results indicate the good damping performance of natural rubber/slide-ring composites for potential applications in isolation rubber bearings.

### Hysteresis tests

**Figure 8:**
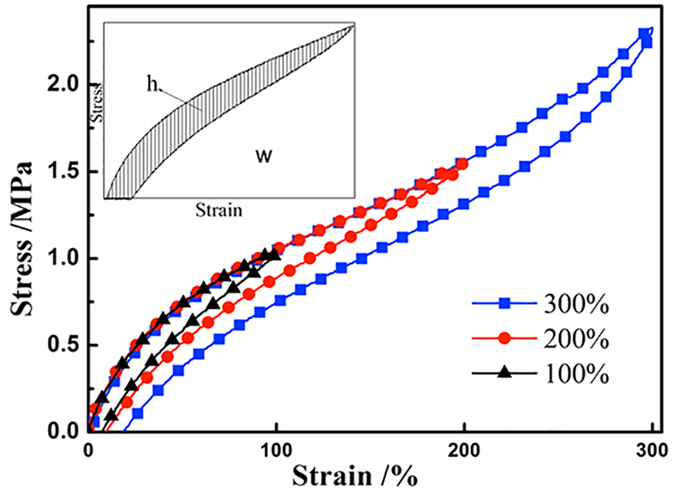
Cyclic stress-strain curves at 100%, 200%, and 300% strains for NR/ENR/SR (70/30/40) composite. Inset: Schematic of cyclic stress-strain curves.

In the hysteresis mode of the tensile tester, the specimens were stretched to 100%, 200%, or 300% strain at a crosshead speed of 1000 mm/min and then allowed to retract at the same rate to the unstretched state. The hysteresis energy density (h) indicates the amount of dissipated energy under cyclic deformation, while the strain energy density (w) indicates the amount of undissipated energy under cyclic deformation (see Fig. 8). The dissipation efficiency (DE), defined as the ability to dissipate energy, is calculated by


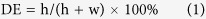


The dissipation efficiency of the natural rubber/slide-ring composites are shown in Table 3. The DE at 100% or 200% strain increases from NR/SR (100/0) to NR/SR (100/50) with the addition of SR, and from NR/ENR/SR (100/0/40) to NR/ENR/SR (70/30/40) with the addition of ENR. The high DE is mainly attributed to the damping properties of the SR and ENR phases at 100% or 200% strain. High DE is essential for isolation rubber bearings. When isolation bearings undergo the equivalent earthquake energy, composites with higher DE are more effective in consuming the earthquake energy.

The DEs of the SR/NR composites are lower than that of NR at 300% strain, because the deformation of SR can not catch up with the deformation of NR. The SR phase are reaching the limits of deformation and losing its ability to dissipate energy. The energy is dissipated mainly by the molecular chains of NR at 300% strain. As for the NR/ENR/SR composites, the ability to dissipate energy of the ENR molecular chains is higher than that of the NR molecular chains at room temperatures causing the existing of epoxy groups. As a result, the same trend of the NR/ENR/SR composites at 300% strain is similar as at 100% and 200% strains.

It is worth noting that the DE values at 200% strain is generally lower than those at 100% and 300% strains. This is caused by the “stress softening” effect of rubber materials. At 100% to 200% strains, the molecular chains in the rubber composites soften and slip easily. There is less interactions to cause the energy dissipation. Therefore, the DE at 200% strain is lower than that at 100% strain. As the strain increasing to 300% strain, orientation of molecular chains and crystallization of the rubber composite occur. More energy is dissipated by NR phase in the emerging and destroying the new orientation and crystallization structures, leading to an improvement in DE at 300% strain.

### Strain-induced crystallization of composites

**Figure 9:**
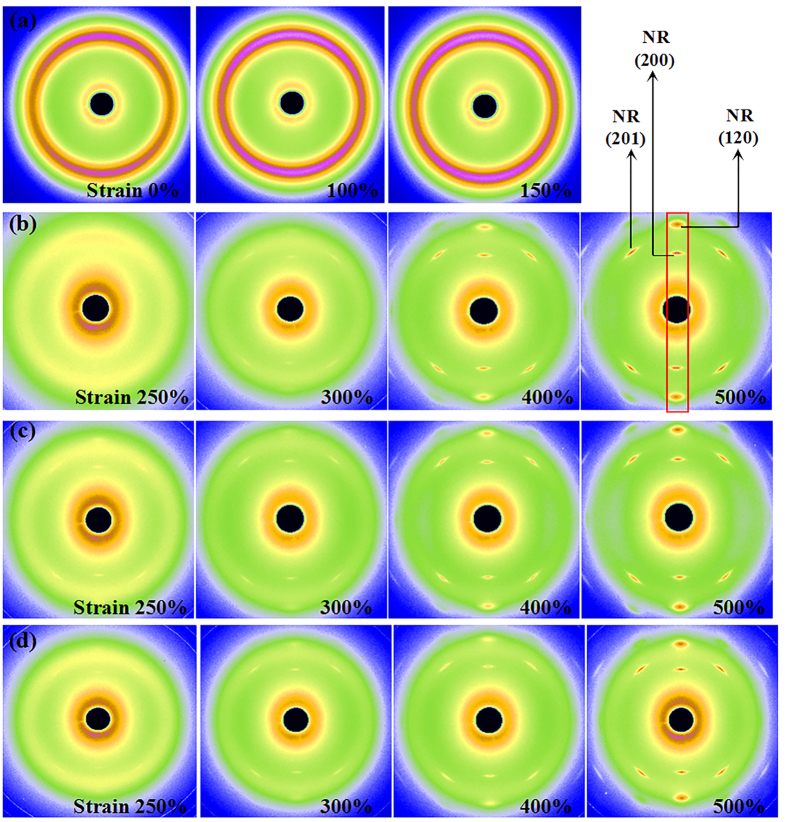
Sequential variation of WAXD patterns for (**a**) pristine SR, (**b**) pristine NR, (**c**) NR/SR (100/40) composite, and (**d**) NR/ENR/SR (80/20/40). Indices of crystallographic planes of NR are indicated in WAXD pattern for NR at 500% strain. Integration region is enclosed by red border.

Figure 9 shows the sequential variation of the WAXD patterns of (a) pristine SR, (b) pristine NR, (c) NR/SR (100/40), and (d) NR/ENR/SR (80/20/40). No crystalline diffraction spot is formed for pristine SR at the maximum strain, indicating that SR is a non-crystallizable polymer. Both NR and NR/SR (100/50) show crystalline diffraction spots in the images. The distinct crystalline diffraction spots of NR first appear at 300% strain for NR and 250% strain for NR/SR (100/50). The addition of SR affects the crystallization of NR. Apparently, the intensities of the crystallographic diffraction spots (120) and (200) planes for NR and NR/SR (100/50) increase with increasing strain.

The WAXD patterns were normalized and integrated along the azimuthal direction within the integration region (red border). The normalized air scattering patterns were also integrated and then used for subtraction. The resultant profiles were deconvoluted into individual indexed peaks and amorphous haloes by using the Levenberg-Marquardt method^7^,^8^,^9^. Each peak on the equatorial diffraction profiles was fitted with a Gaussian function:


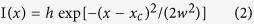


where I(x) is the intensity at position x, and x_c_ refers to the position at the scattering maximum. The parameters h and w indicate the peak height and the peak width, respectively. The degree of crystallization or crystallinity (*X*_*c*_) is estimated by the following equation:


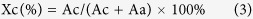


where *A*_*c*_ and *A*_*a*_ are the areas of the crystalline peaks and amorphous halo, respectively.

**Figure 10:**
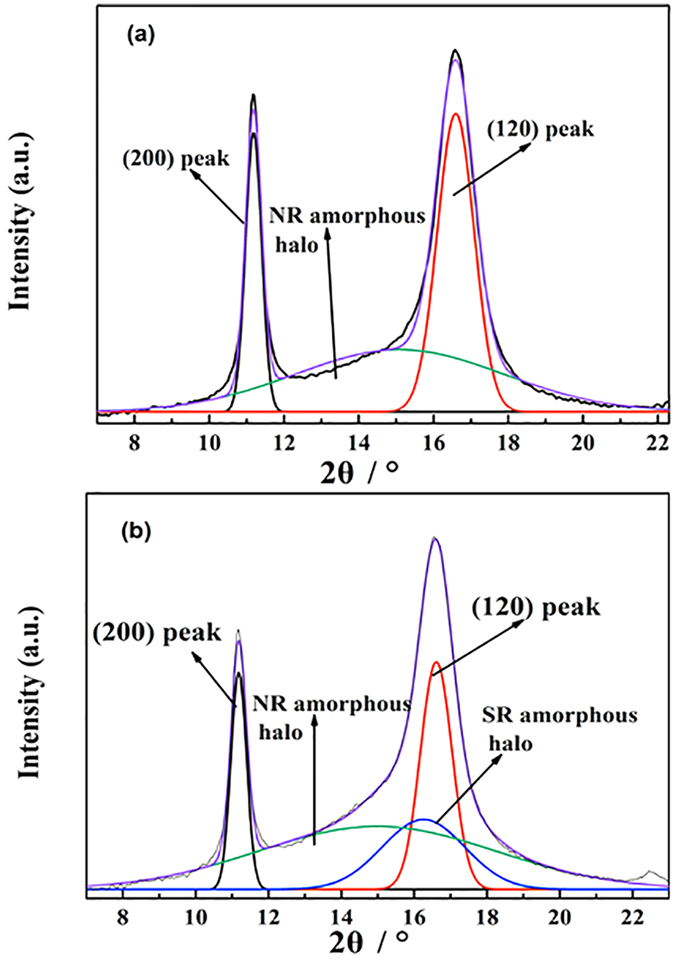
Equatorial diffraction profiles taken from 2D WAXD patterns at 500% strain of (**a**) NR and (**b**) NR/SR (100/50) composite after multi-peak fitting. All curves are normalized and integrated along azimuthal direction.

**Figure 11:**
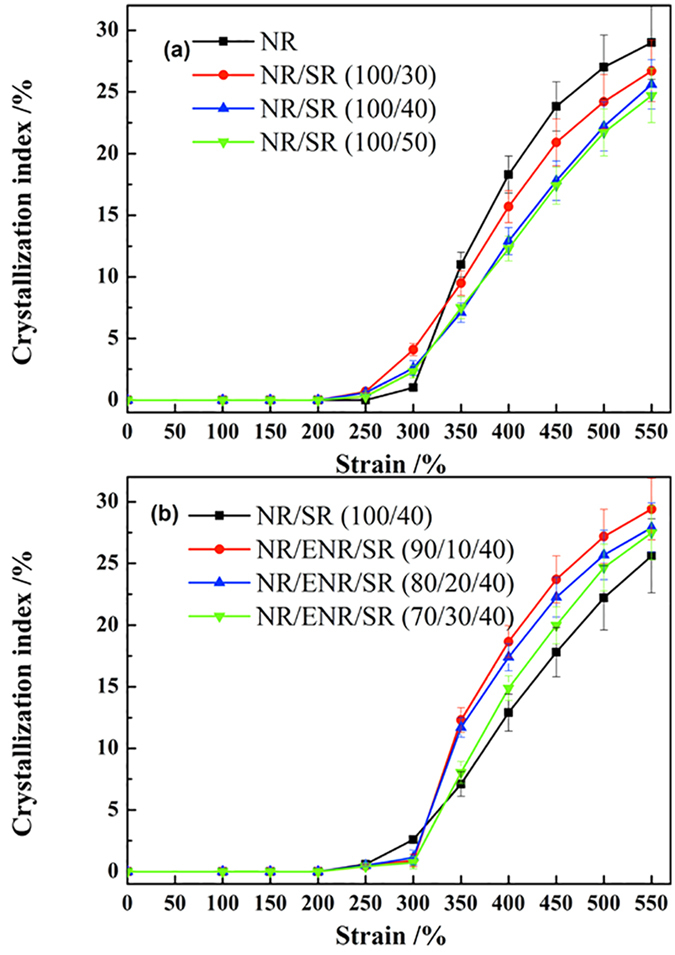
Crystallization index as a function of strain for (**a**) binary NR/SR composites and (**b**) ternary NR/ENR/SR composites.

The equatorial intensity profile of Fig. 9 is shown in Fig. 10. Two diffraction peaks of NR can be clearly observed near 2θ = 11.1 (200) and 2θ = 16.5 (120). The amorphous peaks of NR and SR can also be recognized. In order to monitor the evolution of strain-induced crystallization, the crystallization indices taken from the composites at different strains during stretching are shown in Fig. 11. Figure 11(a) shows the evolution of crystallinity with strain for the NR/SR composites. The results indicate that the onset strains of crystallization are 300% and 250% for NR and the NR/SR composites, respectively, indicating that SR accelerates the crystallization at the preliminary stage of crystallization. In natural rubber, the crosslinks are introduced by chemical bonding, leading to extensive spatial inhomogeneities, which can hinder the strain-induced crystallization of natural rubber by disturbing the orientation process. SR, with “pulley effect”, makes the molecular chains of NR near the interface flexible. These chains tend to orientate more easily at small deformations. The orientated amorphous chains are precursors to the induced crystals. Furthermore, they accelerate the crystallization of NR/SR composites. With strain increasing, the existing of SR phase affect the homogeneities of NR crystallization structure. The destruction of crystalline network will affect the crystallization kinetics and reduce the degree of crystallization eventually.

Figure 11(b) shows the evolution of crystallinity with strain for the NR/ENR/SR composites. The crystallization indices of the NR/ENR/SR (90/10/40) composite at different strain are close to those of pristine NR, and the crystallinity curves of the ternary NR/ENR/SR composites are similar to that of NR. The similar crystallinity curves indicate that the crystallization kinetics of the ternary NR/ENR/SR composites is inherited from the crystallization kinetics of NR. The results indicated that the impact of the SR phase on the crystallization of NR is mitigated by the insulating effect of ENR. As a result, NR and NR/ENR/SR (90/10/40) have the same crystallization index. Since the crystallization of ENR is influenced by the interference of the epoxy functional groups, the crystallinity of ENR is lower than that of pristine NR. With increasing content of ENR, the overall crystallinity of composites decreases.

## Conclusion

The preparation of slide-ring material/natural rubber composites is simple, consisting of blending the damping phase SR with the NR matrix, with ENR acting as the compatibilizer. The high mechanical properties of NR are maintained in these slide-ring material/natural rubber composites. The SR phase and ENR phase can significantly improve the damping factor at room temperatures and the dissipation efficiency of the composites. The impact of the SR phase on the crystallization of NR is mitigated by the insulating effect of ENR. These NR/ENR/SR composites are expected to be potential materials for high damping isolation rubber bearings.

## Additional Information

**How to cite this article**: Wang, W. *et al.* Novel Slide-Ring Material/Natural Rubber Composites with High Damping Property. *Sci. Rep.* **6**, 22810; doi: 10.1038/srep22810 (2016).

## Figures and Tables

**Figure 1:**
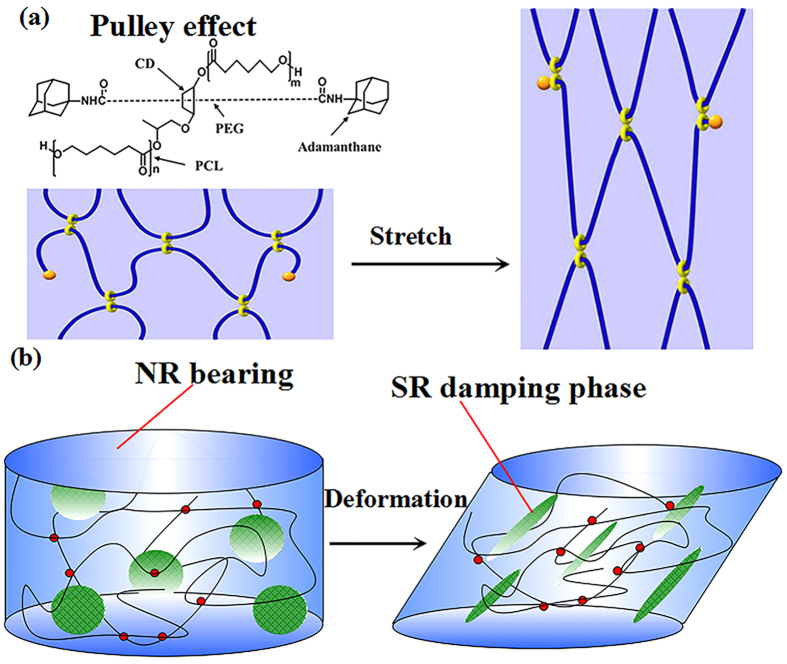
(**a**) “Pulley effect” illustration of slide-ring materials^25^ and (**b**) design concept of high damping natural rubber/slide-ring composites.

**Table 1:** Compounding formulation for natural rubber/slide-ring composites.

Ingredients	Contents/phr^a^
NR + ENR^b^	100
SR	30, 40, or 50
ZnO	3
Stearic acid	1
CZ^c^	0.5
DM^d^	0.5
TMTD^e^	0.2
Sulfur	1.5

^a^phr stands for parts per 100 parts of rubber by weight.

^b^Epoxy degree of ENR is 50%.

^c^N-Cyclohexyl-2-benzothiazole.

^d^2,2′-Dibenzothiazole disulfide.

^e^Tetramethyl thiuram disulfide.

**Table 2:** Tensile properties of NR, SR, and natural rubber/slide-ring composites.

Test	NR	SR	NR/SR (100/30)	NR/SR (100/40)	NR/SR (100/50)	NR/ENR/SR (90/10/40)	NR/ENR/SR (80/20/40)	NR/ENR/SR (70/30/40)
Tensile Modulus at 100% strain/MPa	0.71 ± 0.04	0.01 ± 0.002	0.43 ± 0.03	0.37 ± 0.04	0.34 ± 0.04	0.39 ± 0.03	0.41 ± 0.03	0.38 ± 0.03
Tensile Modulus at 300 strain/MPa	1.83 ± 0.12	0.04 ± 0.005	1.03 ± 0.09	0.84 ± 0.06	0.84 ± 0.07	0.96 ± 0.05	1.00 ± 0.05	0.95 ± 0.07
Tensile strength /MPa	21.6 ± 0.62	0.045 ± 0.007	15.4 ± 0.41	12.5 ± 0.39	12.1 ± 0.36	13.2 ± 0.37	13.3 ± 0.33	12.9 ± 0.35
Elongation at break/%	711 ± 5.38	352 ± 3.88	776 ± 5.34	784 ± 5.61	794 ± 5.53	782 ± 5.23	788 ± 5.46	773 ± 5.44
Hardness	42 ± 0.56	15 ± 0.34	30 ± 0.67	28 ± 0.55	25 ± 0.37	29 ± 0.48	30 ± 0.39	31 ± 0.40

**Table 3:** Dissipation efficiency (DE) of slide-ring material/natural rubber composites.

NR/ENR/SR	DE/%^a^		
	100% strain	200% strain	300% strain
100/0/0	4.7 ± 0.21	4.4 ± 0.18	10.6 ± 0.31
100/0/30	5.6 ± 0.17	4.7 ± 0.13	9.9 ± 0.26
100/0/40	5.9 ± 0.16	5.0 ± 0.23	9.5 ± 0.33
100/0/50	8.0 ± 0.28	6.1 ± 0.21	9.2 ± 0.32
90/10/40	6.1 ± 0.19	5.0 ± 0.18	10.3 ± 0.24
80/20/40	6.9 ± 0.17	5.8 ± 0.22	10.6 ± 0.41
70/30/40	7.6 ± 0.31	6.3 ± 0.24	11.7 ± 0.33

^a^The hysteresis test data were the average of the results obtained from five samples under the same conditions.
